# The Genomic Basis of Adaptation to Profundal Habitats Across the Swiss Alpine Whitefish Radiation

**DOI:** 10.1093/gbe/evag242

**Published:** 2026-10-01

**Authors:** David Frei, Ole Seehausen, Philine G D Feulner

**Affiliations:** Department of Fish Ecology and Evolution, Eawag: Swiss Federal Institute of Aquatic Science and Technology, Kastanienbaum, Switzerland; Division of Aquatic Ecology, Institute of Ecology and Evolution, University of Bern, Bern, Switzerland; Department of Fish Ecology and Evolution, Eawag: Swiss Federal Institute of Aquatic Science and Technology, Kastanienbaum, Switzerland; Division of Aquatic Ecology, Institute of Ecology and Evolution, University of Bern, Bern, Switzerland; Department of Fish Ecology and Evolution, Eawag: Swiss Federal Institute of Aquatic Science and Technology, Kastanienbaum, Switzerland; Division of Aquatic Ecology, Institute of Ecology and Evolution, University of Bern, Bern, Switzerland

**Keywords:** *Coregonus*, adaptive radiation, ecological speciation, population genomics, evolutionary ecology

## Abstract

When populations experience heterogeneous environments, multifarious divergent selection may lead to ecological speciation or even adaptive radiation. The underlying genetic architecture of adaptation can be simple or complex and is relevant for predicting evolutionary responses. Whitefish have radiated across Swiss perialpine lakes, and within the radiation, more than 30 species have been taxonomically described. Across Swiss lake systems, several phenotypically similar species (ie ecomorphs) have evolved independently and utilize distinct environmental niches. Profundal species, adapted to spawn in depths below 60 m, have repeatedly evolved in Lakes Thun, Constance, Lucerne, Zug, and Walen. We here analyzed whole-genome resequencing data of all three to six whitefish species of four of these lakes (Thun, Constance, Lucerne, and Walen) to identify genomic signals of parallel profundal adaptation. The results indicate that a small but spread-out fraction of the genome shows signals of parallel differentiation across all four studied lakes, with pairwise tests revealing more genomic regions with evidence of parallel evolution between profundal species when both were pelagic. Consistent with previous work investigating parallel divergence between two different Alpine whitefish ecomorphs, these results suggest a polygenic basis of adaptation, including genotypic redundancy, which makes repeated evolution more likely but results in little repeatability of its genetic basis.

Significance The genetic architecture of adaptation is relevant for predicting evolutionary responses. Alpine whitefish have repeatedly in different lake systems adapted to utilize profundal lake habitats, and this study suggests that the underlying genetic basis of profundal adaptation is mostly different in different lake systems and species and involves many loci spread across the genome, ie is polygenic. Such an architecture is expected to result in little repeatability of genomic regions showing signatures of adaptation but might well have facilitated the repeated evolution of ecologically similar Alpine whitefish species.

## Introduction

Many well-described adaptive radiations exhibit a certain degree of parallelism in the form of parallel phenotypic evolution, resulting in similar but independently evolved traits in closely related lineages (Futuyma 1986; Schluter et al. 2004). Parallel phenotypic evolution in adaptive radiations is often associated with features of the environment (Schluter et al. 2004), and the repeated evolution of similar traits linked to similar environmental characteristics makes a role of natural selection likely (Schluter 1990; Cai et al. 2019). A set of factors, such as genetic constraints and demographic parameters like effective population size, which affect the availability of (shared) genetic variation and the efficiency of selection, influence the likelihood of similar adaptive traits evolving repeatedly.

The number of genes required to produce a given phenotype can vary dramatically (ie genomic dimensionality), and this aspect of the genetic architecture of traits involved in adaptation might determine potential outcomes. In cases where parallel evolution relies on a few specific genes, evolution is highly constrained as it requires changes in specific genes or loci (Blount et al. 2018; Nosil et al. 2024). However, if phenotypes are polygenic, their evolution may be less constrained as many genes additively contribute to the expression of a given trait value (Yeaman 2015; Barghi et al. 2020). Constraints might be further loosened due to redundancies. Genetic redundancy is defined as two genes having the same function, such that if one of these two genes is inactivated, the phenotype is not affected (Nowak et al. 1997). Following this definition, genotypic redundancy has been defined when more than one genotype can produce the same phenotype (Láruson et al. 2020). In consequence, when a similar phenotype evolved repeatedly due to adaptation to parallel ecological challenges, this is not necessarily predicted to result in genomic parallelism or parallel genomic differentiation. As a result, the fraction of the genome that evolves in parallel varies greatly, even though repeated evolution in response to parallel selective pressures is not uncommon (Blount et al. 2018; Bohutínská et al. 2021). Hence, the degree of genomic parallelism underlying a repeatedly evolved phenotype can range from full genomic parallelism (parallel evolution at all variable positions within genes underlying a trait that is evolving in parallel) to the complete lack thereof as a consequence of genotypic redundancy and high genomic dimensionality (ie polygenic architectures). Hence, trait architectures that might make parallel trait evolution more likely may consist of many loci (ie are polygenic), where the trait value does not rely on specific (rare) mutations (ie genotypic redundancy) or order of mutations; however, such trait architectures result in little repeatability of the underlying genetic basis (Nosil et al. 2024). Furthermore, it has been suggested that simple genetic architectures, with one or few loci of large effect underlying an adaptive phenotype, may facilitate local adaptation or the evolution of adaptive polymorphisms but will not lead to speciation unless niches are geographically isolated and populations breed in the same habitat where they feed (Seehausen et al. 2014; Yeaman 2022). Polygenic architectures, with many loci of small effect additively determining an adaptive phenotype, on the other hand, may more likely facilitate speciation as a by-product (Nosil et al. 2017; Kautt et al. 2020).

Within the nine large perialpine lakes or lake systems of Switzerland, more than 30 Alpine whitefish species have evolved since deglaciation (∼10,000 to 15,000 years BP), with up to six species per lake (Steinmann 1950; Hudson et al. 2011; Selz et al. 2020). In some of the Swiss lakes, phenotypically and ecologically similar species have evolved independently, implying a fundamental role of selection in the process of adaptation and speciation in the Alpine whitefish radiation (Østbye et al. 2005; Hudson et al. 2011). A pattern also replicated in whitefish in Norway, showing parallel morphological divergence between profundal and littoral whitefish (Præbel et al. 2013) and very similar to species-for-species matching (Schluter 1990) as described in *Anolis* lizards (Mahler et al. 2013).

In several (but not all) of these deep perialpine lakes (eg Lakes Constance, Lucerne, Zug, Thun, and Walen), species adapted to the profundal zones of these lakes have independently evolved (Hudson et al. 2011; De-Kayne et al. 2022). Either profundal species are adapted to feed on benthic macroinvertebrates (eg *Coregonus gutturosus* in Lake Constance or *Coregonus profundus* in Lake Thun), or they are adapted to feed on pelagic zooplankton (eg *Coregonus heglingus* in Lake Walen, *Coregonus nobilis* and *Coregonus muelleri* in Lake Lucerne, or *Coregonus albellus* in Lake Thun) (Steinmann 1950; Vonlanthen et al. 2012; Selz et al. 2020; Selz and Seehausen 2023). Deep- and shallow-water habitats of a lake have contrasting environmental characteristics (eg mean and variation in temperature, light conditions, pressure difference, oxygen availability, food resources, parasites, and predation), presumably resulting in multifarious divergent selection between the two habitats (Seehausen and Magalhaes 2010; Ingram et al. 2012). As a result of adaptation to deep water, profundal species are phenotypically differentiated (see the Materials and Methods section for a description of the studied profundal species) from other sympatric species (Steinmann 1950; Doenz et al. 2018; Selz et al. 2020; De-Kayne et al. 2022; Frei et al. 2022; Selz and Seehausen 2023). Often, they are also genetically strongly differentiated from other sympatric species (Ingram et al. 2012; Doenz et al. 2018; De-Kayne et al. 2022; Frei et al. 2022). Because of the high level of genetic differentiation and the clear phenotypic differences, the repeated evolution of profundal whitefish species provides an excellent opportunity to study the genomic landscape of parallel evolution.

Recent work suggests that genomic differentiation underlying ecomorph divergence among sympatric Alpine whitefish species (large-bodied, shallow-spawning benthivores vs. smaller, deeper-spawning planktivores) is scattered across the genome, largely based on a high number of small-effect loci and only very few loci with larger effects (De-Kayne et al. 2022). Likewise, a habitat shift into the profundal zone includes changes in multiple traits (eg body shape, growth rate, spawning time and depth, pigmentation, and eye size) (Steinmann 1950; Hudson et al. 2017; Doenz et al. 2018; Selz et al. 2020; Selz and Seehausen 2023). Thus, the parallel evolution of profundal whitefish species within the Alpine whitefish radiation is probably multidimensional, consisting of changes in multiple traits. Additionally, the genomic basis of (some of) those traits might be genomically multidimensional, ie polygenic, and may contain genotypic redundancy.

During the last century, 29% of the Alpine whitefish species went extinct as a result of anthropogenic eutrophication of many Swiss lakes (Vonlanthen et al. 2012). Profundal species were heavily affected by the hypoxic conditions in deep water during the period of eutrophication that prevented successful reproduction on deep-water spawning grounds and resulted in several extinction events (Vonlanthen et al. 2012; Frei et al. 2022; Selz and Seehausen 2023). Therefore, understanding the genomic basis of profundal adaptation is instrumental to assess the loss of genomic variation, species, and functional diversity caused by anthropogenic eutrophication during the last century.

## Results

### Whole-Genome Sequencing Dataset

Here, we generated whole-genome resequencing data for 91 Alpine whitefish and combined it with data from 78 individuals sequenced in previous studies (De-Kayne et al. 2022; Frei et al. 2022, 2023, 2024) to compile a comprehensive dataset for all taxonomically described whitefish species for four Swiss lakes with one to two profundal-spawning whitefish species in each of them (Table 1; Table S1). These are Lake Thun (six species total, two profundal-spawning species), Lake Lucerne (six species total, two profundal-spawning species), Lake Constance (four species total, one extinct profundal-spawning species), and Lake Walen (three species total, one profundal-spawning species). Because both profundal species of Lake Zug are extinct (Selz and Seehausen 2023) and no suitable tissue samples of these two species were available, we could not include the radiation from Lake Zug. Our full dataset contains 19 species and a total of 169 specimens with an average depth of coverage of about 13.4× (details for each specimen are given in Table S1). This dataset also includes the extinct profundal-spawning *C. gutturosus*, sequenced from scale samples taken in 1937 or 1948 (average depth of coverage 3.9×). Past studies showed that this data can cautiously be integrated with contemporary datasets (Frei et al. 2022, 2024). Across all individuals, base quality values were above 30 (average 37.3, SD 3.4), and mapping quality values were above 29 (average 42.6, SD 4.3; details for each specimen are given in Table S1).

**Table 1:** Overview of the dataset

Lake	Species	Profundal	Diet	Ecomorph	*n*
Thun	*C. profundus*	Yes	Benthic	Benthic profundal	11
	*C. albellus*	Yes	Pelagic	Albeli	9
	*C. acrinasus*	No	Pelagic	Large pelagic	9
	*Coregonus fatioi*	No	Benthic/pelagic	Felchen	9
	*C. steinmanni*	No	Benthic	Balchen	9
	*C. alpinus*	No	Benthic	Balchen	9
Lucerne	*C. nobilis*	Yes	Pelagic	Pelagic profundal	9
	*C. muelleri*	Yes	Pelagic	Albeli	9
	*Coregonus suspensus*	No	Pelagic	Large pelagic	9
	*C. intermundia*	No	Benthic/pelagic	Felchen	9
	*C.* sp. *Alpnacherfelchen*	No	Benthic/pelagic	Felchen	9
	*C. litoralis*	No	Benthic	Balchen	9
Constance	*C. gutturosus*	Yes (extinct)	Benthic	Benthic profundal	10
	*Coregonus wartmanni*	No	Pelagic	Large pelagic	6
	*Coregonus macrophthalmus*	No	Benthic/pelagic	Felchen	10
	*C. arenicolus*	No	Benthic	Balchen	7
Walen	*C. heglingus*	Yes	Pelagic	Albeli	9
	*C. zuerichensis*	No	Benthic/pelagic	Felchen	5
	*C. duplex*	No	Benthic	Balchen	12

For each lake, all species are listed and characterized as profundal spawning or not, by a predominantly benthic or pelagic diet, and categorized into ecomorphs following De-Kayne et al. (2022). The last column gives the number of individuals with whole-genome sequence information for each species.

### Population Structure Within and Between Lakes

To visualize the population structure within and between Lakes Constance, Walen, Lucerne, and Thun, we performed a Principal Component Analysis (PCA) (Fig. 1) based on genotype likelihoods in PCAngsd on our full dataset (3,374,468 Single Nucleotide Polymorphisms [SNPs]). Each lake formed its own cluster in the PC space of principal components one and two, with the exception of *Coregonus acrinasus* from Lake Thun that clustered with the Lake Constance species flock, consistent with earlier work (Hudson et al. 2011; Doenz et al. 2018; Selz et al. 2020; De-Kayne et al. 2022). Although the PCA suggests that several individuals were misidentified in the field and likely belong to another species (see, eg *Coregonus zuerichensis* of Lake Walen or *Coregonus steinmanni* and *C. acrinasus* in Lake Thun), almost all individuals of each of the six studied profundal species (*C. profundus*, *C. albellus*, *C. gutturosus*, *C. nobilis*, *C. muelleri*, and *C. heglingus*) clustered together, respectively, and hence were correctly assigned to their species (see also Figs. S1 to S4 for a separate PCA on each of the four lakes). The exception is one *C. muelleri* specimen (28441), which we excluded from the following differentiation and genome scan analyses. Furthermore, not all species form well distinct genetic cluster; especially, the Felchen ecomorphs often overlap with other species.

**Fig. 1.**
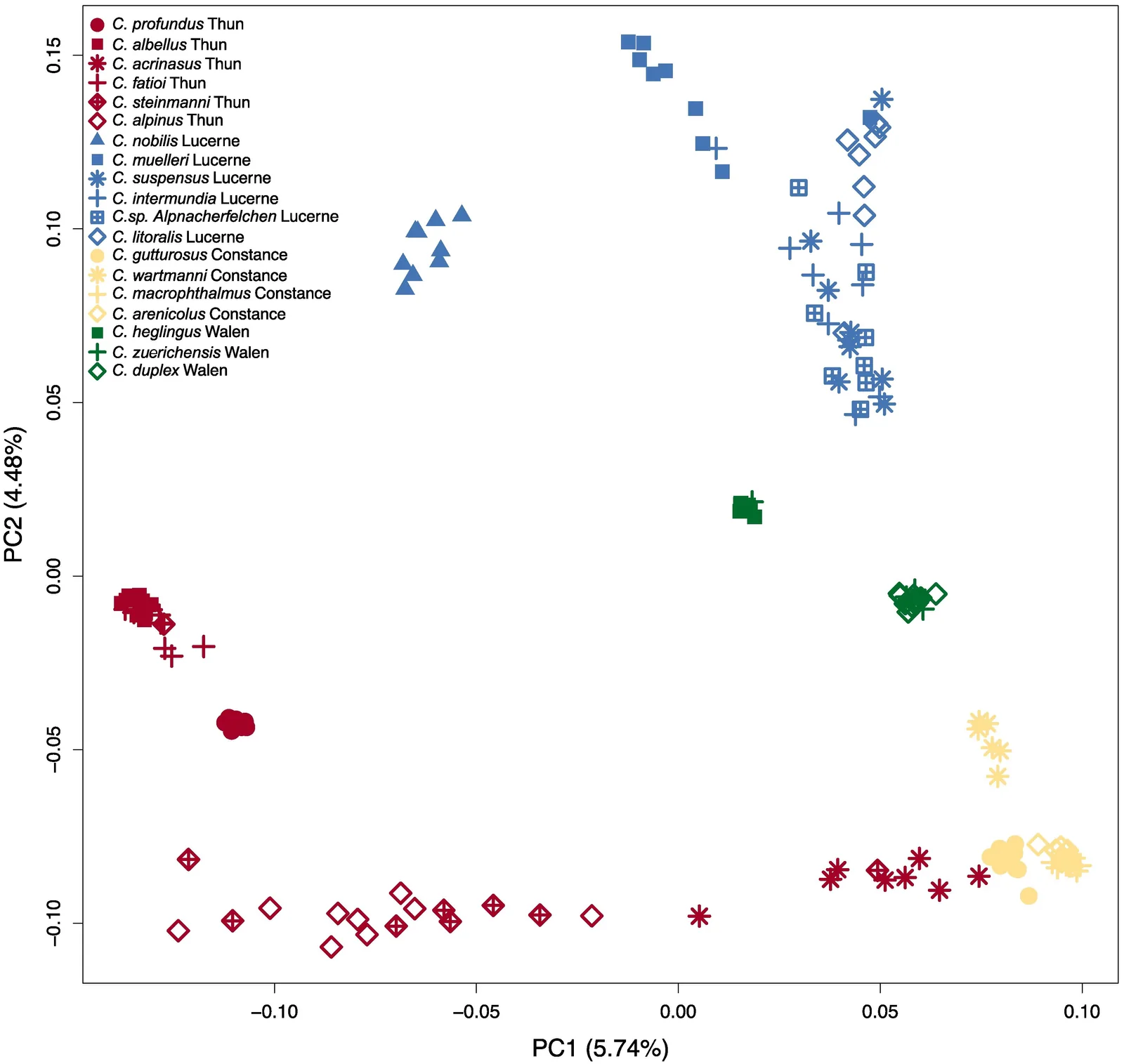
Population structure across all four studied lakes. PCA based on genotype likelihoods of 3,374,468 SNPs and including all sampled lakes and individuals. The plot illustrates the differentiation between lakes (different colors; see legend) and among species (different symbols). Profundal ecomorphs are represented with filled symbols; the same symbols across lakes reflect shared ecomorphs across lakes (see Table 1).

Using a thinned dataset (SNPs being at least 10 kb apart and sites containing at least one homozygous individual) comprising 76,131 SNPs, we generated a maximum likelihood phylogeny including all sampled lakes and species using RAxML-NG (Fig. 2). Our phylogeny, based on a population dataset, does not strongly support species flocks of each lake as monophyletic but is still in line with previous work suggesting monophyletic lake-specific species flocks (Hudson et al. 2011; De-Kayne et al. 2022). It identifies the one known exception *C. acrinasus* of Lake Thun, which clusters within the Lake Constance radiation, consistent with a hybrid origin following translocation of some whitefish from Lake Constance (Doenz et al. 2018; De-Kayne et al. 2022). The monophyly of the Lake Walen species flock is well supported (bootstrap support 88%), while all other deeper nodes in the tree are less well supported. However, bootstrap support toward the tips supporting species improves and is well aligned with species clustering as revealed in the PCA. Especially, both benthic profundal species (*C. gutturosus* in Lake Constance and *C. profundus* in Lake Thun) are well supported (100%) as reciprocally monophyletic. Across all lakes, species of the Balchen ecomorph are well supported as distinct from profundal species, except for one *C. muelleri* specimen that clusters with *Coregonus litoralis*, as already indicated in the PCA and which we excluded as potentially misidentified from the following differentiation analysis. As already noted in the PCA, species of the Felchen ecomorph are often not well supported as monophyletic, and one *Coregonus intermundia* Felchen from Lake Lucerne rather clusters with the Lake Walen branch.

**Fig. 2.**
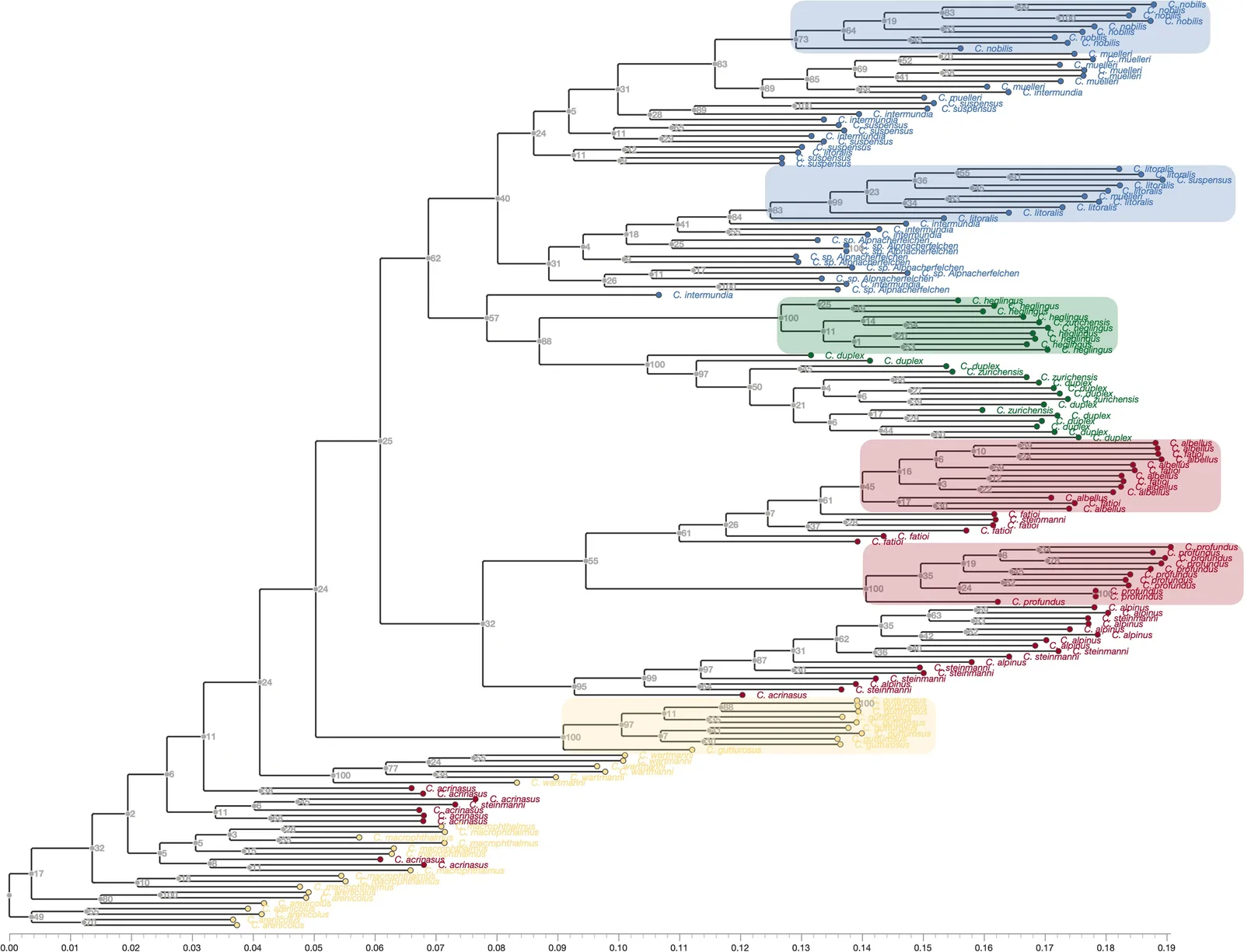
Phylogenetic tree including all individuals and constructed from a thinned dataset (76,131 sites). Tip labels indicate species and are color coded by lake. Branches with profundal species are highlighted by the respective lake color. Note that *C. acrinasus* is a hybrid species between Lake Constance and Lake Thun whitefish following a translocation from Lake Constance into Lake Thun (Doenz et al. 2018; Selz et al. 2020; De-Kayne et al. 2022). Nodes indicate bootstrap support values. The *C. albula* outgroup is not shown but was used to root the tree.

### Genomic Parallelism of Profundal Adaptation

To assess the degree of parallel differentiation between the profundal species in each lake, we calculated *F*_ST_ per position and in 5 kb windows between the profundal species and a sympatric shallow-spawning species of the Balchen ecomorph. Along a water-depth gradient, the Balchen ecomorph represents the opposite extreme to the profundal ecomorphs and is present in all lakes. Genome-wide *F*_ST_ between *C. profundus* and *Coregonus alpinus* in Lake Thun was 0.137, *F*_ST_ between *C. albellus* and *C. alpinus* in Lake Thun was 0.097, *F*_ST_ between *C. nobilis* and *C. litoralis* in Lake Lucerne was 0.096, *F*_ST_ between *C. muelleri* and *C. litoralis* in Lake Lucerne was 0.084 (after exclusion of two potentially misidentified specimens not clustering well in PCA and tree; see Materials and Methods for details), *F*_ST_ between *C. gutturosus* and *Coregonus arenicolus* in Lake Constance was 0.049, and *F*_ST_ between *C. heglingus* and *Coregonus duplex* in Lake Walen was 0.088. In total, only two chromosomal regions (15 out of all 313,054 5 kb windows; 7 windows on chromosome 29 between position 32,970,000 and position 33,080,000; and 8 on chromosome 34 between 38,030,000 and 38,270,000) were in the top fifth percentile in all six *F*_ST_ comparisons between profundal and a sympatric Balchen ecomorph across all four lakes (Fig. 3) and hence indicated parallel differentiation. Only the region on chromosome 29 overlaps with a single gene (maker-PGA_scaffold30__165_contigs__length_48446552-snap-gene-330.3), which is associated with 30 gene ontology (GO) terms, including one molecular function (MF) “protein–hormone receptor activity” and a broad spectrum of potential biological processes (BPs) (Table S2). Besides this rather restrictive way of looking for regions in the genome evidential of parallel evolution, we also used PicMin. PicMin is derived from the theory of order statistics and tests for repeated molecular evolution, estimating significance at the level of an individual locus, using the results of genome scans, such as *F*_ST_ scans (Booker et al. 2023). The statistical power to detect repeated adaptation at the genome level increases with the number of lineages that have signals of repeated adaptation of a given locus (ie genomic window) (Booker et al. 2023).

**Fig. 3.**
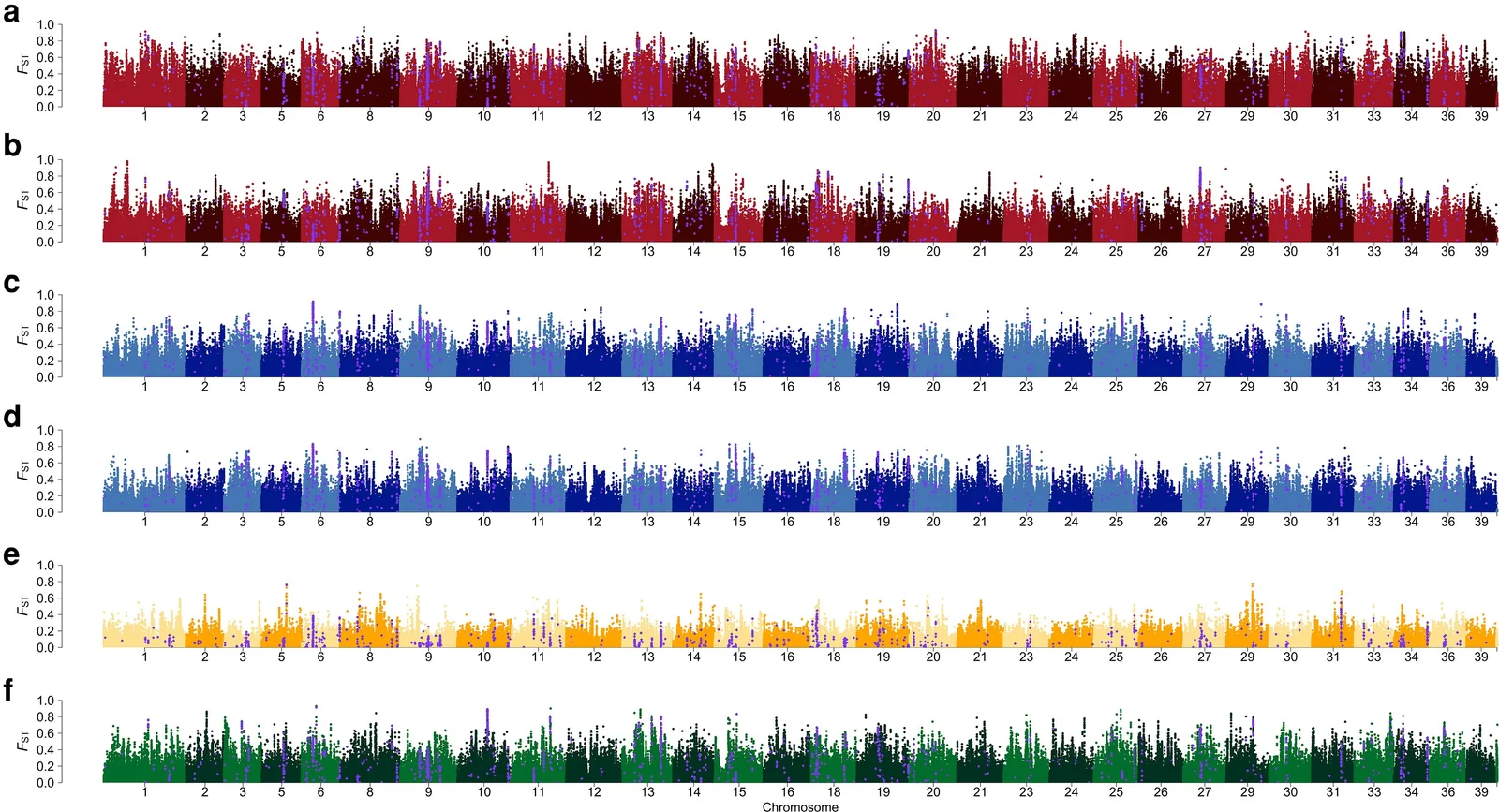
Genomic differentiation of profundal species along the genome. *F*_ST_ comparisons between profundal and a sympatric shallow-spawning species of the Balchen ecomorph in each lake along the genome in 5 kb windows. a) *C. profundus* against *C. alpinus* Lake Thun. b) *C. albellus* against *C. alpinus* Lake Thun. c) *C. nobilis* against *C. litoralis* Lake Lucerne. d) *C. muelleri* against *C. litoralis* Lake Lucerne. e) The extinct *C. gutturosus* against *C. arenicolus* Lake Constance. f) *C. heglingus* against *C. duplex* Lake Walen. Purple diamonds indicate outlier windows with evidence of parallel evolution as indicated by a PicMin analysis. Chromosomes indicated as potentially collapsed duplicated regions in the reference genome have been excluded.

Utilizing the strength of the PicMin analysis by comparing across six *F*_ST_ contrasts between four sympatric shallow-spawning species of the Balchen ecomorph and the six profundal species, ∼0.43% (1,304 out of 313,054 windows) of all genomic windows showed parallel elevated *F*_ST_ signals (Fig. 3, purple diamonds, and Fig. 4; Fig. S5). A PicMin analysis across only the four pelagic profundal contrasts identified parallel elevated *F*_ST_ signals in 1,485 windows, ∼0.47% of all windows. In the 1,304 outlier windows identified across all six profundal species, on average, four profundal species shared an elevated *F*_ST_ signal, and across all six profundal species, Tajima's D values were significantly reduced in those outlier windows compared to the rest of the genome, consistent with expectations of parallel adaptive evolution (Tajima's D decline ranged between 0.10 and 0.35, with confidence intervals that did not overlap 0 for any of the six species; a one-sided permutation test confirmed a significant decline in all six cases, *P* < 0.001). We identified 222 genes that overlapped outlier windows (*q* < 0.05) and used those for the functional enrichment analysis. This set of genes was enriched for several GO terms (Table S3), including the terms “hypoxia-induced intrinsic apoptotic signaling pathway” and “stress-induced mitochondrial fusion,” among other terms related to stress or immune responses as well as terms associated with neural and bone development.

**Fig. 4.**
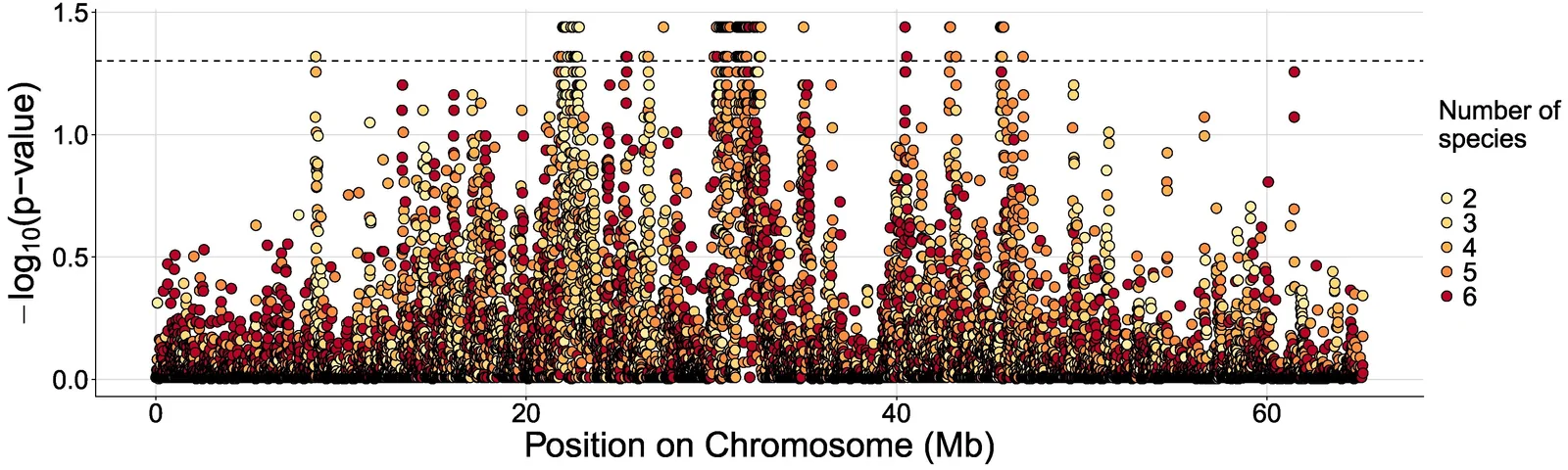
Genomic parallelism in profundal adaptation across the Alpine whitefish radiation. PicMin analysis including all six profundal species in all four lakes identifying outlier windows with evidence for parallel elevated *F*_ST_ signals. The plot shows the results along chromosome 9 of the reference genome (the chromosome with the most evidence for parallel evolution, containing 148 of all 1,304 outlier windows showing evidence for parallel evolution; other chromosomes are shown in Fig. S5). The plot shows fdr-corrected *P* values of 5 kb windows along the genome. Color corresponds to the number of species, and warmer red colors indicate more species support the evidence for parallel evolution. The dashed line indicates the significance threshold (*q* < 0.05).

In addition, the two-way PicMin (Booker et al. 2023) analysis based on the *F*_ST_ between the profundal species and the respective sympatric Balchen ecomorph in each lake revealed significantly more windows indicative of parallel genomic differentiation than expected by chance in all pairwise comparisons (Fig. 5). All resulting *P* values were highly significant (see Table S4). Interestingly, the number of windows with parallel signals varied across comparisons. All five comparisons with two pelagic profundal species had more shared outlier windows than any of the seven comparisons comparing benthic and profundal species. The study system only allows for one comparison of two benthic species, which held the fewest shared windows.

**Fig. 5.**
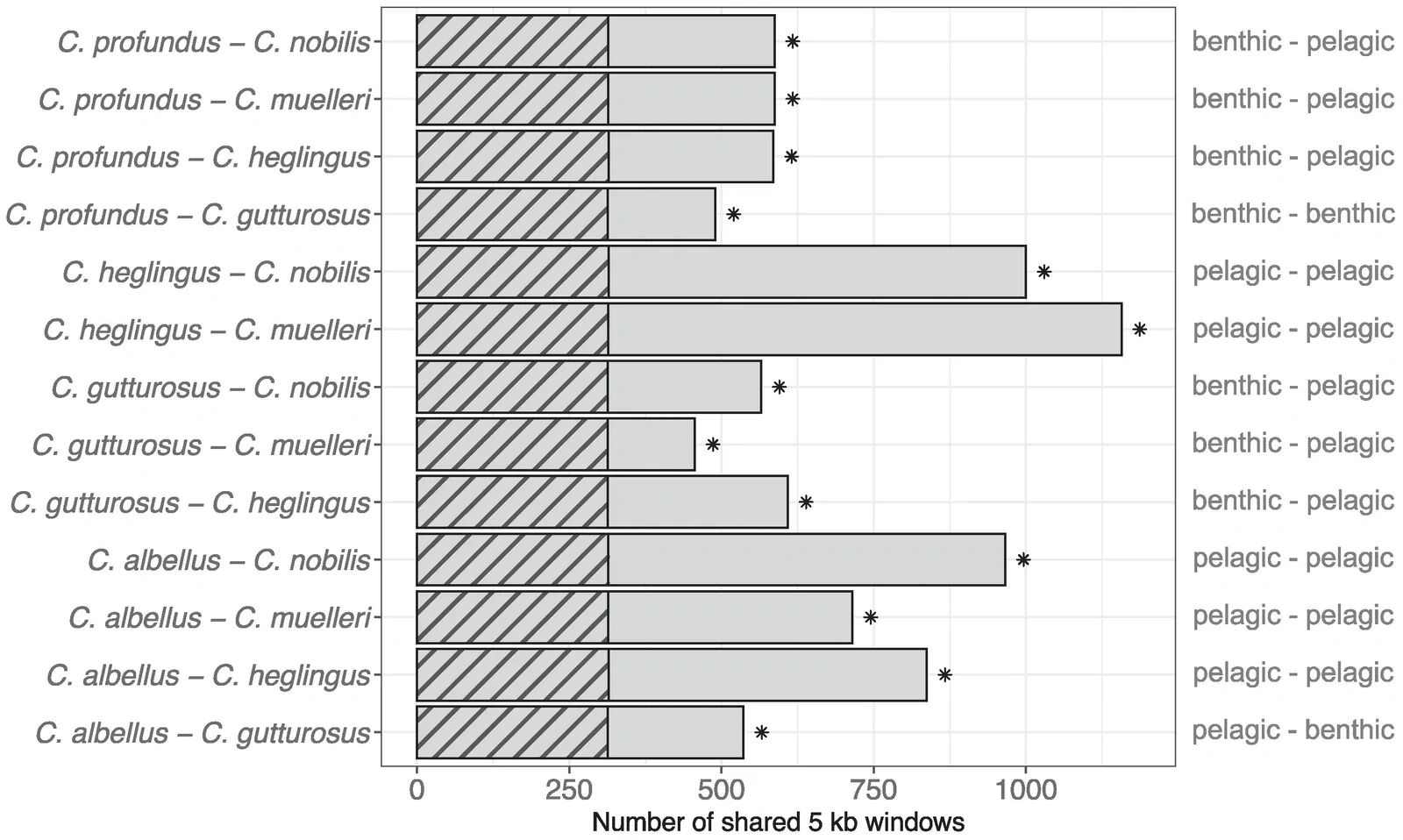
Pairwise signals of parallel genomic differentiation of profundal species. Barplots showing the results of the pairwise PicMin analysis. Bars show the observed number of windows with signals of parallel differentiation for all possible pairwise comparisons of profundal species. The two comparisons within lakes (*C. nobilis–C. muelleri* in Lake Lucerne, *C. albellus–C. profundus* in Lake Thun) are not shown due to the biological and statistical dependence of those comparisons. The hatched area corresponds to the null expectation for the number of shared differentiated windows by chance, and in all comparisons, significantly more windows were detected as differentiated in parallel (for *P* values, see Table S4).

## Discussion

Parallel evolution is a common phenomenon between well-described adaptive radiations, resulting in similar but independently evolved phenotypes in different species (Futuyma 1986; Schluter et al. 2004). Within the postglacial Alpine whitefish radiation, several profundal whitefish species have evolved independently in different lake-specific subradiations since deglaciation. We generated whole-genome resequencing data of all species from four lakes (and subradiations) that contain at least one profundal species to test for genomic signals of parallel evolution. Our results reveal little parallel genomic differentiation but overall suggest a polygenic basis of divergence and adaptation, including considerable genotypic redundancy.

The six studied profundal species are recovered mostly as monophyletic species in our phylogenetic reconstruction, a pattern that not all other species show. Rather, both the PCA and the phylogenetic tree suggest that sympatric species vary in their degree of clustering and lineage sorting, which could suggest different stages of speciation (Nosil et al. 2009). The Alpine whitefish radiation is evolutionarily young, and there is evidence both for ancestral (Hudson et al. 2011; De-Kayne et al. 2022) and recent hybridization (Vonlanthen et al. 2012; Frei et al. 2022). Such hybridization might affect genetic clustering and lineage sorting and might revert the process of speciation (ie speciation reversal) (Taylor et al. 2006). Previous work specifically indicated genomic evidence for noticeable hybridization between profundal species with sympatric sister species during a period of anthropogenic eutrophication (Feulner and Seehausen 2019; De-Kayne et al. 2022) and the transfer of profundal genomic variation during such speciation reversal (Frei et al. 2022). Hence, the monophyletic clustering of profundal species seems remarkable. However, we here study profundal species sampled before speciation reversal (Lake Constance) (Frei et al. 2022) and three lakes (Thun, Walen, Lucerne) that have been shown to be less affected by anthropogenic eutrophication and speciation reversal (Vonlanthen et al. 2012). The clear genomic clustering and the long branches subtending some of the profundal species in our phylogenetic tree might suggest that the profundal species we study here might have progressed further in their speciation process compared to some of their sympatric sister species. For example, many species of the Felchen ecomorph that spawn at intermediated depths seem less distinct. Pronounced differences between two environments can result in strong divergent selection (Nosil 2012), and divergent ecological selection can contribute to barriers to gene flow (Schluter 2000; Rundle and Nosil 2005). Hence, a pronounced ecological contrast such as experienced at the end of a depth continuum and the resulting divergent selection between the profundal and other species might have contributed to barriers to gene exchange and a pronounced progression along the speciation continuum.

Closely related lineages are predicted to exhibit a higher level of genomic parallelism than distantly related lineages because they are more likely to share the same ancestral polymorphisms (Bohutínská et al. 2021). Thus, a high degree of parallelism in the genomic basis of profundal adaptation might be expected, as the Alpine whitefish radiation is evolutionarily young and little time had passed between separation into lake lineages and the radiations in each lake unfolding. In contrast to this prediction, we identified only a relatively small number (∼0.43%) of windows along the genome showing signals of parallel evolution across all comparisons. However, all pairwise tests for genomic parallelism between all possible combinations of profundal species resulted in highly significant *P* values. Comparisons including two profundal pelagic species (*C. albellus*, *C. heglingus*, *C. muelleri*, and *C. nobilis*) resulted in a higher number of genomic windows with signals of parallel evolution than any other comparisons. This suggests that the degree of genomic parallelism between the four profundal pelagic whitefish species is higher than between the two profundal benthic species (*C. gutturosus* and *C. profundus*) and higher than that between pelagic and benthic profundals. This might indicate that niche multidimensionality plays a role during species divergence, as various and potentially distinct traits (such as for pelagic or benthic feeding) have been selected in different profundal species across replicated depth gradients. Overall, genomic parallelism in profundal adaptation across the four studied perialpine lake radiations seems rather low. In other systems with repeated adaptation to shared environmental contrasts, eg the marine periwinkle *Littorina saxatilis*, much higher proportions of the genome (2% to 28%) show signals of parallel evolution (Westram et al. 2014; Ravinet et al. 2016; Morales et al. 2019).

Several factors might have contributed to such a comparatively low extent of genomic parallelism across lakes but highly significant genomic parallelism in pairwise comparisons between lakes. Genome scan approaches like the one used here assume that demography has the same effect on all loci across the genome, but that genomic regions showing an exceptionally strong differentiation between species are indicative of being shaped by divergent selection and/or being shielded from homogenization by gene flow (Wu 2001; Turner et al. 2005; Michel et al. 2010). However, the neutral variance in *F*_ST_ values can be increased by populations’ demography and sampling design choices (Bierne et al. 2013; Fourcade et al. 2013; Lotterhos and Whitlock 2014, 2015; Lou et al. 2021). This might cause randomly distributed false positives in individual scans for increased genomic differentiation and hinder the detection of repeated occurrences of the same regions of increased differentiation in several independently evolved population or species contrasts. However, the detection of repeated occurrences of increased differentiation in the same genomic regions has been suggested as difficult to explain by neutral processes alone (Yeaman 2013). Furthermore, genome features like variation in recombination rate and structural variants, such as inversions and duplications, might also alter variance in *F*_ST_ values locally (Feulner et al. 2013; Roesti et al. 2013; Booker et al. 2020) and, in case those features are shared across contrasts, might cause repeated occurrences of elevated differentiation, which falsely might be interpreted as genomic regions experiencing parallel evolution. Hence, it might be particularly relevant that the ancestor of the salmonid lineage experienced a whole-genome duplication 80 to 100 million years ago (Near et al. 2012; Macqueen and Johnston 2014; Lien et al. 2016). The genomes of the different salmonid lineages were uniquely shaped during the rediploidization that happened independently within each lineage following this salmonid-specific fourth vertebrate whole-genome duplication (Robertson et al. 2017). Hence, this whole-genome duplication event could have been a source of redundancy in coregonines. De-Kayne et al. (2022) indeed identified two sets of paralogs that underlie an ecomorph contrast in the Alpine whitefish radiation. Studies in North American whitefish species pairs similarly found evidence for low levels of genomic parallelism together with indications of genotypic redundancy (Renaut et al. 2011; Bernatchez et al. 2016). In addition, ecologically relevant traits might have a highly polygenic basis within the Alpine whitefish radiation (De-Kayne et al. 2022). A polygenic basis likely includes genotypic redundancy and results in little parallelism at the molecular level (Pritchard et al. 2010; Barghi et al. 2020; Nosil et al. 2024). However, such a polygenic architecture with genotypic redundancy holds the potential to facilitate rapid and repeated adaptation, as there are multiple genotypic solutions to similar ecological challenges, reducing potential constraints. In addition, multifarious divergent selection, which is likely at play here given that multiple environmental variables change with depth, such as temperature, light conditions, pressure, oxygen availability, food resources, parasites, and predation, has been suggested to promote diversification and speciation (Nosil et al. 2009). This effect might be due to a greater number of traits involved (Nosil et al. 2009) or potentially be mainly mediated by an increased number of loci affected by genomic dimensionality per se (ie polygenic architecture of traits) (White and Butlin 2021). Genomic simulations with varying trait architecture suggested that genotypic redundancy can facilitate adaptation along multivariate environmental gradients, such as in climate change scenarios (Terasaki Hart and Wang 2024), which might find a spatial analog in water-depth gradients. Hence, when environmental gradients are geographically replicated (eg in different lakes), multifarious divergent selection on multidimensional traits with a polygenic architecture and genotypic redundancy might facilitate rapid and repeated adaptation; however, genomic parallelism would be expected to be limited. Thus, such polygenic adaptation is relevant in the context of adaptive radiations due to its capacity to facilitate adaptation and diversification processes, but also in the context of environmental change because of its potential to facilitate rapid evolutionary responses to changing environmental conditions.

## Materials and Methods

### Study System

In all four studied lakes (Thun, Constance, Lucerne, and Walen), one or two profundal-spawning whitefish species have been described, although the species of Lake Constance went extinct during the period of anthropogenic eutrophication (Vonlanthen et al. 2012; Frei et al. 2022, 2023). However, due to a historical scale sample collection, sequencing data of this extinct species has been published (Frei et al. 2022). The six profundal-spawning species used in this study are either benthos feeders (*C. gutturosus* in Lake Constance and *C. profundus* in Lake Thun; Steinmann 1950; Vonlanthen et al. 2012; Selz et al. 2020) or they are pelagic zooplanktivores (*C. heglingus* in Lake Walen, *C. nobilis* and *C. muelleri* in Lake Lucerne, and *C. albellus* in Lake Thun; Selz et al. 2020; Selz and Seehausen 2023).

Lake Thun harbors two profundal-spawning species: *C. profundus* and *C. albellus*. *C. profundus* and *C. albellus* are both slow-growing species (Steinmann 1950; Vonlanthen et al. 2012; Selz et al. 2020). Both species spawn during summer and fall (and sometimes winter) at depths below 60 m (Doenz et al. 2018). *C. profundus* has a very low gill-raker count, presumably as a result of adaptation to feeding on benthic macroinvertebrates from soft sediment, while *C. albellus* exhibits a high gill-raker count, presumably as a result of adaptation to feeding on zooplankton in the water column (Vonlanthen et al. 2012; Selz et al. 2020).

The extinct Lake Constance species *C. gutturosus* is phenotypically similar to *C. profundus* of Lake Thun. *C. gutturosus* was slow growing and spawned during summer and winter in deep regions of the lake (Steinmann 1950). The species exhibited a very low gill-raker count and was feeding exclusively on benthic macroinvertebrates (Steinmann 1950; Vonlanthen et al. 2012).

*C. nobilis* and *C. muelleri* in Lake Lucerne also spawn in profundal regions of the lake (Vonlanthen et al. 2012; Selz and Seehausen 2023). *C. nobilis* is described as a summer-spawning species, while *C. muelleri* has a long spawning period with peaks during late summer and winter (Vonlanthen et al. 2012; Selz and Seehausen 2023). Both *C. nobilis* and *C. muelleri* have a high gill-raker count, which is characteristic of species that feed on zooplankton (Vonlanthen et al. 2012; Selz and Seehausen 2023).

*C. heglingus* in Lake Walen is ecologically very similar to *C. albellus* in Lake Thun and *C. muelleri* in Lake Lucerne. *C. heglingus* is a slow-growing species and spawns in the profundal zone of Lake Walen (Vonlanthen et al. 2012). Both summer- and winter-spawning populations have been described (Vonlanthen et al. 2012). *C. heglingus* has a high gill-raker count and feeds on zooplankton (Vonlanthen et al. 2012).

### Sample Collection

The samples that were sequenced here were collected for previous studies (Hudson et al. 2017) for Lake Lucerne (Doenz et al. 2018), for Lake Thun, and (Selz et al. 2025) for Lake Walen, in accordance with permits issued by the cantons of Zurich and St. Gallen (ZH128/15) for Lake Walen and Lake Constance, Bern (BE68/15) for Lake Thun, and Lucerne (LU04/14) for Lake Lucerne. Individual specimens were phenotypically assigned to species. Individuals were anesthetized and subsequently euthanized using appropriate concentrations of tricaine methane sulfonate solutions (MS-222). Finclips were taken and stored in 100% analytical ethanol until extraction of DNA, and muscle tissue was preserved frozen at −80°C. DNA extraction was done using the Qiagen DNeasy blood and tissue kit (Qiagen AG, CH) following the manufacturer’s recommendations.

### DNA Extraction and Sequencing

For each sample where a finclip was available, one Illumina paired-end TruSeq DNA PCR-Free library (Illumina GmbH, CH) was prepared. For muscle tissue samples, an Illumina paired-end TruSeq DNA Nano library (Illumina GmbH, CH) was produced (see Table S1 for an overview of all sequenced samples; 19 species, 5 to 12 individuals per species). Library preparation was performed by the Next Generation Sequencing (NGS) platform of the University of Bern following the manufacturer's instructions. Libraries were sequenced on a NovaSeq 6000 S4 flow cell at the NGS platform of the University of Bern. We additionally used existing sequencing data generated by De-Kayne et al. (2022)—downloaded from European Nucleotide Archive (ENA) with accession PRJEB47792—and data generated by Frei et al. (2022, 2023, 2024), downloaded from ENA under accessions PRJEB43605, PRJEB53050, and PRJEB57733.

### Processing Reads and Mapping

Raw sequencing reads were processed according to Frei et al. (2022). In brief, Poly-G strings were removed using fastp 0.20.0 (Chen et al. 2018), and overlapping paired-end reads with a length longer than 25 bp were merged using SeqPrep version 1.0 (https://github.com/jstjohn/SeqPrep). Raw reads were aligned to the Alpine whitefish genome assembly (ENA accession: GCA_902810595.1) (De-Kayne et al. 2020) with bwa mem version 0.7.12 (Li et al. 2009) and default parameters, except for adjusting the “r” parameter to 1 (increasing the accuracy of alignment but reducing computational speed). Duplicated reads were marked with MarkDuplicates, mate information was fixed with FixMateInformation, and read groups were replaced with AddOrReplaceReadGroups using Picard tools (version 2.20.2; http://broadinstitute.github.io/picard/). We excluded all chromosomes of the Alpine whitefish reference genome indicated as potentially collapsed duplicated regions during the assembly process, as described in De-Kayne et al. (2020).

### Population Genomic Analysis

Genotype likelihoods and minor allele frequencies at polymorphic sites were calculated using the samtools genotype likelihood model in ANGSD 0.925 (Li 2011; Korneliussen et al. 2014). We only included sites covered with at least two reads in each of at least 159 individuals (of a total of 169 individuals, resulting in a maximum of 6% missing data), passing a *P*-value cutoff of 10E−6 for being variable and having no more than two different alleles. Reads that did not map uniquely, those with a mapping quality below 30, and bases with a quality score below 20 were excluded from the calculation of genotype likelihoods. The following *P*-value cutoffs for SNP filters in ANGSD version 0.925 were used: -sb_pval 0.05 -qscore_pval 0.05 -edge_pval 0.05 -mapq_pval 0.05. This resulted in a total of 8,182,760 polymorphic sites for further analysis.

We then used a minor allele frequency threshold of 5% to perform a PCA on the whole dataset using PCAngsd 1.02 (Meisner and Albrechtsen 2018), resulting in a total of 3,374,468 SNPs. In addition, we split the dataset by lake and subsequently performed a PCA using PCAngsd 1.02, applying a 5% minor allele frequency threshold.

To identify genomic regions where the profundal species of each lake are distinct, we contrasted the profundal-spawning species of each lake with a sympatric shallow-spawning species of the Balchen ecomorph of the respective lake. In most lakes, profundal and Balchen ecomorphs cluster well into distinct groups in the PCA and on the phylogenetic tree and/or are well separated from each other; however, in Lake Lucerne, it appears that one profundal and one Balchen individual might have been misidentified, so we excluded those from the genome scan analysis (*C. muelleri* specimen 28441 and *C. litoralis* specimen 28597). We calculated the weighted genome-wide *F*_ST_, the weighted *F*_ST_ per SNP, and the *F*_ST_ in nonoverlapping 5 kb windows along the genome in ANGSD 0.940 based on 1D and 2D site frequency spectra inferred from site allele frequencies based on genotype likelihoods (Nielsen et al. 2012; Bhatia et al. 2013; Korneliussen et al. 2014, 2013). We first assessed the strict overlap between the 5 kb windows with the highest differentiation (top 5% quantile) in R (R Core Team 2018). We then further used *F*_ST_ in 5 kb windows along the genome between each profundal species and a sympatric, shallow-spawning Balchen species to identify signals of parallel differentiation and, ie parallel adaptation to profundal habitats, using the R-package PicMin 0.0.0.9 (Booker et al. 2023). We followed the vignette of the package on GitHub (https://github.com/TBooker/PicMin/blob/main/vignettes/Arabidopsis-vignette.Rmd) and adjusted the threshold to determine adaptation to 0.05 (which is a suitable threshold for four lineages; Booker et al. 2023). We performed the analysis on all six profundal-spawning species combined (number of lineages = 6). Because our aim was to screen the genome for signals of repeated differentiation, we used a relatively inclusive rather than a restrictive approach with a significance threshold of *q* < 0.05 (Booker et al. 2023), following Bohutínska et al. (2021). In addition, we assessed parallel differentiation separately for each pairwise combination of the six studied profundal species with the PicMin approach (see https://github.com/TBooker/PicMin/blob/main/vignettes/Arabidopsis-vignette.Rmd).

To check if outlier windows as identified by PicMin for showing parallel elevation of *F*_ST_ also reveal signatures of selection, we calculated Tajima's D for all six profundal species in 5 kb along the genome using ANGSD 0.935. Fitting a linear fixed-effect model using lmer in R, we checked if outlier windows showing parallel elevation of *F*_ST_ indicate a decline of Tajima's D values in comparison to nonoutlier windows, a pattern suggestive of containing an excess of rare alleles in the focal profundal species, consistent with positive selection. The model fitted chromosome as a random effect, and we estimated CIs. CIs not overlapping zero were interpreted as a significant reduction in Tajima's D, which was further tested by a permutation approach (10,000 permutations, one-sided test). Windows that indicated significant signals of parallel evolution by PicMin across all profundal species were further investigated to identify which genes they overlap with. As suggested by Booker et al. (2023), we used a permissive significance threshold (*q* > 0.05) for this purpose to maximize the number of genes for the enrichment analysis. Gene annotations (from the Alpine whitefish genome (De-Kayne et al. 2020); ENA accession: GCA_902810595.1) that overlap in their position with the identified windows indicating signals of parallel evolution were identified using bedtools 2.28.0 (Quinlan 2014). Gene enrichment for specific GO terms (from https://datadryad.org/stash/dataset/doi:10.5061/dryad.xd2547ddf) within these windows was tested using the R package topGO 2.38.1 (Alexa et al. 2006) separately for each of the three ontology classes: cellular component, BPs, and MF. We used Fisher's exact test, applying the “weight” algorithm to each ontology class (with no fdr multiple testing correction in accordance with the topGO manual). The ten most significant hits for each ontology class were reported.

To visualize phylogenetic relationships between lakes as well as between species within each lake, we produced a maximum likelihood phylogeny. We calculated genotype likelihoods using the whole dataset of 169 individuals and including the *Coregonus albula* outgroup from De-Kayne et al. (2022) (ENA sample accession ERS11527697) with ANGSD 0.925 (Korneliussen et al. 2014). We again used the same parameters as above (data of minimally 159 individuals at each position, at least two reads from every individual, a *P*-value cutoff of 10E−6 for being variable, minimum mapping quality of 30 and minimum base quality of 20, only two alleles, and we used the identical *P*-value cutoffs for SNP filters), resulting in a total of 14,546,220 SNPs. We then inferred genotypes and phased these using beagle 4.1 (Browning and Browning 2007). We thinned this dataset using VCFtools 0.1.16 (Danecek et al. 2011) so that all SNPs were at least 10 kb apart from each other and then filtered the resulting dataset with bcftools 1.10.2 (https://github.com/samtools/bcftools) to contain only sites that are homozygous for the reference, and homozygous for the alternative allele in at least one individual, resulting in a total of 76,131 SNPs. We then converted the VCF- to a phylip file using the python script vcf2phylip.py (https://github.com/edgardomortiz/vcf2phylip). We used RAxML-NG 1.01 (Kozlov et al. 2019) to produce the phylogeny with the GTR+G+ASC_LEWIS substitution model and default parameters and 100 bootstrap runs. The resulting phylogeny was plotted with PearTree 1.2.2 (https://github.com/artic-network/peartree/releases/tag/v1.2.2).

## Supplementary Material

evag242_Supplementary_Data

## Data Availability

The raw sequencing files are accessible on ENA Sequence Read Archive (SRA) (project accession PRJEB96847). Additional supporting data are deposited on the eawag research data institutional collection (DOI: https://doi.org/10.25678/000F98).
