# Supplementary material for: The Genomic Basis of Adaptation to Profundal Habitats Across the Swiss Alpine Whitefish Radiation
Source: Genome Biol Evol. 2026 Oct 1;18(10):evag242. doi: 10.1093/gbe/evag242 (PMC13689696; doi:10.1093/gbe/evag242)
Supplement: Supplementary file 1 [file evag242_supplementary_data.docx]

Supplementary Information

**Figure S1: PCA on all individuals from Lake Thun.** PCA based on genotype likelihoods of 3’125’823 SNPs polymorphic sites and including all individuals of Lake Thun. The plot illustrates the differentiation amongst species (plotting symbol according to species, see legend within the plot; profundal species represented by filled symbols).

**Figure S2: PCA on all individuals from Lake Constance.** PCA based on genotype likelihoods of 3’261’099 SNPs polymorphic sites and including all individuals of Lake Constance. The plot illustrates the differentiation amongst species (plotting symbol according to species, see legend within the plot; the profundal species represented by filled symbols).

**Figure S3: PCA on all individuals from Lake Walen.** PCA based on genotype likelihoods of 3’099’149 SNPs polymorphic sites and including all individuals of Lake Walen. The plot illustrates the differentiation amongst species (plotting symbol according to species, see legend within the plot; the profundal species represented by filled symbols).

**Figure S4: PCA on all individuals from Lake Lucerne.** PCA based on genotype likelihoods of 3’237’140 SNPs polymorphic sites and including all individuals of Lake Lucerne. The plot illustrates the differentiation amongst species (plotting symbol according to species, see legend within the plot; profundal species represented by filled symbols).

**Supplementary Figure 5: Parallel evolution along the genome.** Manhattan plots showing the PicMin analysis including all four lakes along all chromosomes. The plot shows fdr-corrected p-values of 5 kb windows along the genome. Colour corresponds to number of species and warmer red colours indicate more species support the evidence for parallel evolution. The dashed line shows the significance threshold (q < 0.1) Chromosomes indicated as potentially collapsed duplicated regions in the reference genome have been excluded.

**Supplementary Table 1:** **Overview over all sequenced samples.** Table shows the species assignment, the lab ID number, the lake where the sample has been collected, for which study the sample has been sequenced, tissue type that was used, and sequencing data statistics (mean depth of coverage, mean base and mapping quality).

| **Species** | **Lab ID** | **Lake** | **Sequenced by** | **Tissue** | **meandepth** | **meanbaseq** | **meanmapq** |
| --- | --- | --- | --- | --- | --- | --- | --- |
| *C. acrinasus* | 123105 | Thun | this study | Finclip | 10.9 | 35.6 | 43.5 |
| *C. steinmanni* | 123117 | Thun | this study | Finclip | 14.2 | 35.1 | 43.5 |
| *C. acrinasus* | 123118 | Thun | this study | Finclip | 9.3 | 35.7 | 43.8 |
| *C. alpinus* | 123131 | Thun | this study | Finclip | 11.8 | 35.2 | 43.9 |
| *C. acrinasus* | 123149 | Thun | this study | Finclip | 8.8 | 35.7 | 43.9 |
| *C. steinmanni* | 123178 | Thun | this study | Finclip | 11.8 | 35 | 43.7 |
| *C. acrinasus* | 123201 | Thun | this study | Finclip | 10.5 | 35.6 | 43.3 |
| *C. acrinasus* | 123202 | Thun | this study | Finclip | 9.1 | 35.6 | 43.2 |
| *C. alpinus* | 123231 | Thun | this study | Finclip | 11 | 34.9 | 43.9 |
| *C. steinmanni* | 123233 | Thun | this study | Finclip | 13.2 | 35.1 | 43.9 |
| *C. albellus* | 123245 | Thun | this study | Finclip | 8.6 | 35.8 | 43.2 |
| *C. acrinasus* | 123263 | Thun | this study | Finclip | 7.9 | 35.9 | 43.0 |
| *C. steinmanni* | 123264 | Thun | this study | Finclip | 13.6 | 35 | 42.9 |
| *C. alpinus* | 123276 | Thun | this study | Finclip | 11.2 | 35.4 | 43.6 |
| *C. steinmanni* | 123288 | Thun | this study | Finclip | 10 | 35.2 | 43.4 |
| *C. fatioi* | 123500 | Thun | this study | Finclip | 9 | 35.2 | 44.4 |
| *C. alpinus* | 123631 | Thun | this study | Finclip | 13.1 | 35.2 | 44 |
| *C. profundus* | 123679 | Thun | this study | Finclip | 13.3 | 35 | 43.4 |
| *C. profundus* | 123688 | Thun | this study | Finclip | 14.9 | 35.2 | 43.4 |
| *C. profundus* | 123689 | Thun | this study | Finclip | 9.4 | 35.7 | 43.5 |
| *C. albellus* | 123838 | Thun | this study | Finclip | 9 | 35.2 | 44.4 |
| *C. fatioi* | 123840 | Thun | this study | Finclip | 12.1 | 35.2 | 44.1 |
| *C. fatioi* | 123841 | Thun | this study | Finclip | 14.2 | 35.2 | 43.3 |
| *C. albellus* | 123885 | Thun | this study | Finclip | 10.6 | 35 | 44.4 |
| *C. fatioi* | 123936 | Thun | this study | Finclip | 10.6 | 35.3 | 43.4 |
| *C. albellus* | 123953 | Thun | this study | Finclip | 9.7 | 35.1 | 44.1 |
| *C. albellus* | 123954 | Thun | this study | Finclip | 10.4 | 35 | 43 |
| *C. albellus* | 123960 | Thun | this study | Finclip | 8.6 | 35.3 | 43.4 |
| *C. profundus* | 123964 | Thun | this study | Finclip | 9.9 | 35.1 | 43.3 |
| *C. profundus* | 123966 | Thun | this study | Finclip | 10 | 35.2 | 42.9 |
| *C. profundus* | 123975 | Thun | this study | Finclip | 10.9 | 35 | 43.2 |
| *C. fatioi* | 123990 | Thun | this study | Finclip | 11.2 | 35.5 | 43.0 |
| *C. profundus* | 124003 | Thun | this study | Finclip | 10.1 | 35.1 | 43.8 |
| *C. steinmanni* | 124025 | Thun | this study | Finclip | 13.6 | 35 | 42.2 |
| *C. alpinus* | 124029 | Thun | this study | Finclip | 11 | 35.1 | 44.3 |
| *C. alpinus* | 124030 | Thun | this study | Finclip | 9.2 | 35.1 | 43.6 |
| *C. fatioi* | 124219 | Thun | this study | Finclip | 12.9 | 35.3 | 44.0 |
| *C. profundus* | 124249 | Thun | this study | Finclip | 8.9 | 35.8 | 43.1 |
| *C. heglingus* | 175718 | Walen | this study | Finclip | 11 | 35.2 | 43.4 |
| *C. heglingus* | 175723 | Walen | this study | Finclip | 12.5 | 35.1 | 43.9 |
| *C. heglingus* | 175725 | Walen | this study | Finclip | 7.4 | 37.3 | 43.4 |
| *C. zurichensis* | 175729 | Walen | this study | Finclip | 10.2 | 35.7 | 43.3 |
| *C. zurichensis* | 175731 | Walen | this study | Finclip | 11.1 | 36.3 | 43.0 |
| *C. heglingus* | 175735 | Walen | this study | Finclip | 9.2 | 35.2 | 43.7 |
| *C. heglingus* | 175738 | Walen | this study | Finclip | 11 | 36 | 42.9 |
| *C. heglingus* | 175742 | Walen | this study | Finclip | 8.6 | 35.8 | 43.4 |
| *C. duplex* | 175801 | Walen | this study | Finclip | 10.4 | 36 | 43 |
| *C. duplex* | 175802 | Walen | this study | Finclip | 9.3 | 36.3 | 42.5 |
| *C. duplex* | 175806 | Walen | this study | Finclip | 9.3 | 35.9 | 43 |
| *C. duplex* | 175807 | Walen | this study | Finclip | 8.5 | 36.6 | 42.6 |
| *C. duplex* | 175809 | Walen | this study | Finclip | 8.1 | 37.2 | 41 |
| *C. zurichensis* | 175856 | Walen | this study | Finclip | 9.8 | 35.7 | 43.2 |
| *C. zurichensis* | 175865 | Walen | this study | Finclip | 8.8 | 35.8 | 43.1 |
| *C. zurichensis* | 175868 | Walen | this study | Finclip | 11.3 | 35.7 | 43.2 |
| *C. duplex* | 175870 | Walen | this study | Finclip | 7.5 | 37 | 41.5 |
| *C. sp. Alpnacherfelchen* | 26003 | Lucerne/Alpnach | this study | Muscle | 8.4 | 37.6 | 42.9 |
| *C. sp. Alpnacherfelchen* | 26005 | Lucerne/Alpnach | this study | Muscle | 7.3 | 37.8 | 42.2 |
| *C. sp. Alpnacherfelchen* | 26007 | Lucerne/Alpnach | this study | Muscle | 7.3 | 36.2 | 42.8 |
| *C. sp. Alpnacherfelchen* | 26009 | Lucerne/Alpnach | this study | Muscle | 8.2 | 37.1 | 42.9 |
| *C. sp. Alpnacherfelchen* | 26010 | Lucerne/Alpnach | this study | Muscle | 7.7 | 37.8 | 42.9 |
| *C. sp. Alpnacherfelchen* | 26017 | Lucerne/Alpnach | this study | Muscle | 7.8 | 37.6 | 42.9 |
| *C. nobilis* | 26819 | Lucerne | this study | Muscle | 6.8 | 38.6 | 41.7 |
| *C. nobilis* | 26832 | Lucerne | this study | Muscle | 6.6 | 37.4 | 43.3 |
| *C. nobilis* | 26836 | Lucerne | this study | Muscle | 6.5 | 37.9 | 42.5 |
| *C. nobilis* | 26841 | Lucerne | this study | Muscle | 7.5 | 38 | 42.3 |
| *C. nobilis* | 26850 | Lucerne | this study | Muscle | 7.5 | 37.5 | 43.5 |
| *C. nobilis* | 26852 | Lucerne | this study | Muscle | 7.6 | 37.9 | 42.7 |
| *C. intermundia* | 28433 | Lucerne | this study | Muscle | 8.9 | 38.0 | 43.2 |
| *C. muelleri* | 28438 | Lucerne | this study | Muscle | 6.5 | 38.3 | 42.9 |
| *C. muelleri* | 28441 | Lucerne | this study | Muscle | 5.8 | 40.5 | 41.2 |
| *C. muelleri* | 28461 | Lucerne | this study | Muscle | 6.4 | 39.2 | 43.1 |
| *C. muelleri* | 28462 | Lucerne | this study | Muscle | 6.4 | 38.9 | 42.5 |
| *C. intermundia* | 28464 | Lucerne | this study | Muscle | 8.4 | 35.8 | 43.8 |
| *C. intermundia* | 28466 | Lucerne | this study | Muscle | 6.6 | 39.2 | 42.1 |
| *C. muelleri* | 28485 | Lucerne | this study | Muscle | 5.7 | 40.5 | 41 |
| *C. muelleri* | 28488 | Lucerne | this study | Muscle | 5.9 | 40.6 | 41.3 |
| *C. litoralis* | 28506 | Lucerne | this study | Muscle | 9.6 | 35.4 | 44.2 |
| *C. litoralis* | 28592 | Lucerne | this study | Muscle | 6.3 | 39 | 42.5 |
| *C. litoralis* | 28593 | Lucerne | this study | Muscle | 7.3 | 37.3 | 43.7 |
| *C. litoralis* | 28595 | Lucerne | this study | Muscle | 8.1 | 35.9 | 43.6 |
| *C. litoralis* | 28597 | Lucerne | this study | Muscle | 1.4 | 41.8 | 40.9 |
| *C. suspensus* | 28611 | Lucerne | this study | Muscle | 9.2 | 37.7 | 42.8 |
| *C. suspensus* | 28615 | Lucerne | this study | Muscle | 8.2 | 37.6 | 43.0 |
| *C. suspensus* | 28632 | Lucerne | this study | Muscle | 6.5 | 37.4 | 42.1 |
| *C. intermundia* | 28646 | Lucerne | this study | Muscle | 5.8 | 39.6 | 41.5 |
| *C. intermundia* | 28647 | Lucerne | this study | Muscle | 8.0 | 37.5 | 43.2 |
| *C. intermundia* | 28681 | Lucerne | this study | Muscle | 6.7 | 38.6 | 41.4 |
| *C. suspensus* | 28714 | Lucerne | this study | Muscle | 7.8 | 37.0 | 43.5 |
| *C. suspensus* | 28715 | Lucerne | this study | Muscle | 8.6 | 37.4 | 43.4 |
| *C. suspensus* | 28720 | Lucerne | this study | Muscle | 7.3 | 38.5 | 43.1 |
| *C. litoralis* | 28733 | Lucerne | this study | Muscle | 4.7 | 41.4 | 37.9 |
| *C. gutturosus* | DF_1 | Constance | Frei et al. 2022 | Scale | 4.6 | 51.8 | 23.9 |
| *C. gutturosus* | DF_4 | Constance | Frei et al. 2022 | Scale | 6.9 | 47.8 | 30.6 |
| *C. gutturosus* | DF_6 | Constance | Frei et al. 2022 | Scale | 10.9 | 48.3 | 30 |
| *C. gutturosus* | DF_8 | Constance | Frei et al. 2022 | Scale | 5.6 | 47.5 | 27.6 |
| *C. gutturosus* | DF_12 | Constance | Frei et al. 2022 | Scale | 3 | 49.1 | 24.3 |
| *C. gutturosus* | S01_0006 | Constance | Frei et al. 2024 | Scale | 1.4 | 50.2 | 27.2 |
| *C. gutturosus* | S01_0020 | Constance | Frei et al. 2024 | Scale | 1.2 | 51.4 | 24.7 |
| *C. gutturosus* | S01_0039 | Constance | Frei et al. 2024 | Scale | 1.4 | 52 | 25 |
| *C. gutturosus* | S20_0051 | Constance | Frei et al. 2024 | Scale | 1.9 | 49.8 | 26.5 |
| *C. gutturosus* | S20_0079 | Constance | Frei et al. 2024 | Scale | 2.2 | 50.3 | 25.8 |
| *C. arenicolus* | 126 | Constance | Frei et al. 2022 | Finclip | 36.4 | 37.2 | 45 |
| *C. arenicolus* | 127 | Constance | Frei et al. 2022 | Finclip | 34.1 | 39.1 | 41.2 |
| *C. arenicolus* | 128 | Constance | Frei et al. 2022 | Finclip | 27.1 | 38.8 | 41.6 |
| *C. arenicolus* | 212631 | Constance | Frei et al. 2023 | Finclip | 5.4 | 36.4 | 45 |
| *C. arenicolus* | 212633 | Constance | Frei et al. 2023 | Finclip | 5.5 | 36.4 | 44.6 |
| *C. arenicolus* | 212634 | Constance | Frei et al. 2023 | Finclip | 4 | 36.4 | 44.8 |
| *C. arenicolus* | 212701 | Constance | Frei et al. 2023 | Finclip | 3.7 | 36.3 | 44 |
| *C. wartmanni* | 121 | Constance | Frei et al. 2022 | Finclip | 33.9 | 36.8 | 44.6 |
| *C. wartmanni* | 122 | Constance | Frei et al. 2022 | Finclip | 28.1 | 37.3 | 44.3 |
| *C. wartmanni* | 123 | Constance | Frei et al. 2022 | Finclip | 34.4 | 37.1 | 44.4 |
| *C. wartmanni* | 131 | Constance | Frei et al. 2022 | Finclip | 35.8 | 37.2 | 44.7 |
| *C. wartmanni* | 212704 | Constance | Frei et al. 2024 | Finclip | 12.5 | 35.5 | 38.7 |
| *C. wartmanni* | 212705 | Constance | Frei et al. 2024 | Finclip | 10.8 | 35.5 | 44.7 |
| *C. macrophthalmus* | 132 | Constance | Frei et al. 2022 | Finclip | 23.6 | 37.6 | 43.6 |
| *C. macrophthalmus* | 212621 | Constance | Frei et al. 2023 | Finclip | 2.1 | 36.3 | 44.5 |
| *C. macrophthalmus* | 212639 | Constance | Frei et al. 2023 | Finclip | 3.3 | 36.4 | 44.1 |
| *C. macrophthalmus* | 212660 | Constance | Frei et al. 2023 | Finclip | 3.4 | 36.3 | 44.2 |
| *C. macrophthalmus* | 212661 | Constance | Frei et al. 2023 | Finclip | 3.6 | 36.3 | 44.0 |
| *C. macrophthalmus* | 212673 | Constance | Frei et al. 2023 | Finclip | 6.5 | 36.3 | 44.3 |
| *C. macrophthalmus* | 212682 | Constance | Frei et al. 2023 | Finclip | 4.4 | 36.3 | 44.2 |
| *C. macrophthalmus* | 212685 | Constance | Frei et al. 2023 | Finclip | 3.4 | 36.2 | 44.5 |
| *C. macrophthalmus* | 212686 | Constance | Frei et al. 2023 | Finclip | 4.2 | 36.3 | 44.1 |
| *C. macrophthalmus* | 212689 | Constance | Frei et al. 2023 | Finclip | 4.2 | 36.3 | 43.5 |
| *C. acrinasus* | 12 | Thun | De-Kayne et al. 2022 | Finclip | 23.2 | 37.1 | 42.8 |
| *C. acrinasus* | 13 | Thun | De-Kayne et al. 2022 | Finclip | 20.8 | 36.5 | 44.6 |
| *C. acrinasus* | 14 | Thun | De-Kayne et al. 2022 | Finclip | 30.2 | 36.9 | 43.8 |
| *C. alpinus* | 16 | Thun | De-Kayne et al. 2022 | Finclip | 20.1 | 36.2 | 45.3 |
| *C. alpinus* | 17 | Thun | De-Kayne et al. 2022 | Finclip | 22.9 | 36.4 | 45.3 |
| *C. alpinus* | 19 | Thun | De-Kayne et al. 2022 | Finclip | 22.4 | 36.9 | 44.9 |
| *C. fatioi* | 21 | Thun | De-Kayne et al. 2022 | Finclip | 26.4 | 37.1 | 43.8 |
| *C. fatioi* | 22 | Thun | De-Kayne et al. 2022 | Finclip | 30.7 | 36.3 | 45.5 |
| *C. fatioi* | 23 | Thun | De-Kayne et al. 2022 | Finclip | 21.1 | 36.2 | 45.3 |
| *C. profundus* | 26 | Thun | De-Kayne et al. 2022 | Finclip | 23.9 | 36.7 | 43.7 |
| *C. profundus* | 27 | Thun | De-Kayne et al. 2022 | Finclip | 25.7 | 37.1 | 43.6 |
| *C. profundus* | 29 | Thun | De-Kayne et al. 2022 | Finclip | 24.8 | 37 | 43.6 |
| *C. intermundia* | 47 | Lucerne | De-Kayne et al. 2022 | Muscle | 21.8 | 36.0 | 45.4 |
| *C. intermundia* | 48 | Lucerne | De-Kayne et al. 2022 | Muscle | 23.2 | 36.1 | 44.9 |
| *C. intermundia* | 50 | Lucerne | De-Kayne et al. 2022 | Muscle | 26.6 | 36.1 | 45.4 |
| *C. steinmanni* | 56 | Thun | De-Kayne et al. 2022 | Finclip | 16 | 36.4 | 45.3 |
| *C. steinmanni* | 58 | Thun | De-Kayne et al. 2022 | Finclip | 39 | 37 | 44.3 |
| *C. steinmanni* | 59 | Thun | De-Kayne et al. 2022 | Finclip | 17 | 36.3 | 45.3 |
| *C. albellus* | 61 | Thun | De-Kayne et al. 2022 | Finclip | 21.5 | 36.7 | 44.3 |
| *C. albellus* | 63 | Thun | De-Kayne et al. 2022 | Finclip | 21 | 36.2 | 45.2 |
| *C. albellus* | 64 | Thun | De-Kayne et al. 2022 | Finclip | 28.2 | 36.2 | 45.1 |
| *C. sp. Alpnacherfelchen* | 66 | Lucerne/Alpnach | De-Kayne et al. 2022 | Finclip | 18.4 | 36.0 | 44.8 |
| *C. sp. Alpnacherfelchen* | 68 | Lucerne/Alpnach | De-Kayne et al. 2022 | Finclip | 20.3 | 36.1 | 44.8 |
| *C. sp. Alpnacherfelchen* | 70 | Lucerne/Alpnach | De-Kayne et al. 2022 | Finclip | 16.3 | 36.1 | 44.6 |
| *C. duplex* | 76 | Walen | De-Kayne et al. 2022 | Finclip | 23.4 | 37 | 44.7 |
| *C. duplex* | 78 | Walen | De-Kayne et al. 2022 | Finclip | 23.4 | 36.7 | 44.6 |
| *C. duplex* | 79 | Walen | De-Kayne et al. 2022 | Finclip | 21.9 | 36.2 | 45.6 |
| *C. heglingus* | 81 | Walen | De-Kayne et al. 2022 | Finclip | 21.6 | 36.9 | 45.1 |
| *C. heglingus* | 82 | Walen | De-Kayne et al. 2022 | Finclip | 23.6 | 36.6 | 45.4 |
| *C. heglingus* | 83 | Walen | De-Kayne et al. 2022 | Finclip | 31.8 | 36.7 | 45.1 |
| *C. duplex* | 86 | Walen | De-Kayne et al. 2022 | Finclip | 23.6 | 36.7 | 45.3 |
| *C. duplex* | 87 | Walen | De-Kayne et al. 2022 | Finclip | 24 | 36.5 | 45.5 |
| *C. duplex* | 90 | Walen | De-Kayne et al. 2022 | Finclip | 25 | 36.9 | 44.8 |
| *C. nobilis* | 103 | Lucerne | De-Kayne et al. 2022 | Muscle | 17 | 36.3 | 44.7 |
| *C. nobilis* | 104 | Lucerne | De-Kayne et al. 2022 | Muscle | 20.6 | 36.2 | 45.2 |
| *C. nobilis* | 106 | Lucerne | De-Kayne et al. 2022 | Muscle | 25.3 | 36.3 | 44.8 |
| *C. suspensus* | 107 | Lucerne | De-Kayne et al. 2022 | Muscle | 17.4 | 36.2 | 44.9 |
| *C. suspensus* | 108 | Lucerne | De-Kayne et al. 2022 | Muscle | 20.6 | 36.2 | 45.2 |
| *C. suspensus* | 109 | Lucerne | De-Kayne et al. 2022 | Muscle | 21.5 | 36.3 | 45.0 |
| *C. muelleri* | 110 | Lucerne | De-Kayne et al. 2022 | Muscle | 32.1 | 36.2 | 45.1 |
| *C. muelleri* | 111 | Lucerne | De-Kayne et al. 2022 | Muscle | 23.8 | 36.3 | 45.3 |
| *C. muelleri* | 113 | Lucerne | De-Kayne et al. 2022 | Muscle | 23.6 | 36.3 | 45.5 |
| *C. litoralis* | 114 | Lucerne | De-Kayne et al. 2022 | Muscle | 26.4 | 36.2 | 44.7 |
| *C. litoralis* | 118 | Lucerne | De-Kayne et al. 2022 | Muscle | 20.6 | 36.3 | 44.6 |
| *C. litoralis* | 119 | Lucerne | De-Kayne et al. 2022 | Muscle | 28.6 | 36.3 | 44.8 |

**Supplementary Table 2:** **GO terms associated with the single gene (maker-PGA_scaffold30__165_contigs__length_48446552-snap-gene-330.3) showing parallel differentiation across all comparisons.** The first column shows the unique GO identifier, the second column gives the respective terminological description, and the last column gives one of the three ontologies of interest (CC cellular component, BP biological process, MF molecular function).

| **GO.ID** | **Term** | **Class** |
| --- | --- | --- |
| GO:0016500 | protein-hormone receptor activity | MF |
| GO:0042700 | luteinizing hormone signaling pathway | BP |
| GO:0005886 | plasma membrane | CC |
| GO:0004930 | G protein-coupled receptor activity | BP |
| GO:0016021 | membrane | CC |
| GO:0009986 | cell surface | CC |
| GO:0042699 | follicle-stimulating hormone signaling pathway | BP |
| GO:0005623 | obsolete cell | CC |
| GO:0060408 | regulation of acetylcholine metabolic process | BP |
| GO:0001545 | primary ovarian follicle growth | BP |
| GO:0008584 | male gonad development | BP |
| GO:0009992 | intracellular water homeostasis | BP |
| GO:0035093 | sperm DNA condensation | BP |
| GO:0045779 | negative regulation of bone resorption | BP |
| GO:0033148 | positive regulation of intracellular estrogen receptor signaling pathway | BP |
| GO:0060065 | uterus development | BP |
| GO:0010640 | regulation of platelet-derived growth factor receptor signaling pathway | BP |
| GO:0071711 | basement membrane organization | BP |
| GO:0045670 | regulation of osteoclast differentiation | BP |
| GO:0003073 | regulation of systemic arterial blood pressure | BP |
| GO:0007200 | phospholipase C-activating G protein-coupled receptor signaling pathway | BP |
| GO:0007193 | adenylate cyclase-inhibiting G protein-coupled receptor signaling pathway | BP |
| GO:0050673 | epithelial cell proliferation | BP |
| GO:0007626 | locomotory behavior | BP |
| GO:0002064 | epithelial cell development | BP |
| GO:0043408 | regulation of MAPK cascade | BP |
| GO:0031175 | neuron projection development | BP |
| GO:0033044 | regulation of chromosome organization | BP |
| GO:0038194 | thyroid-stimulating hormone signaling pathway | BP |
| GO:0030728 | ovulation | BP |

**Supplementary Table 3:** **Functional enrichment of windows with signals of parallel evolution.** GO enrichment analysis for all windows that showed signals of parallel evolution in our PicMin analysis including all four lakes. The first column shows the unique GO identifier for each enriched term, the second column gives the respective terminological description, the third column gives the number of genes annotated with the term within the genome, the fourth column gives the number how often the term was represented within windows with a signature of positive selection, the fifth column gives how often the term was expected in those windows by chance, the next column give the p-value using Fisher’s exact method based on gene counts, and the last column gives one of the three ontologies of interest (CC cellular component, BP biological process, MF molecular function) that have been explored.

| **GO.ID** | **Term** | **Annotated** | **Significant** | **Expected** | **P-value** | **Class** |
| --- | --- | --- | --- | --- | --- | --- |
| GO:0070836 | caveola assembly | 10 | 2 | 0.06 | 0.0016 | BP |
| GO:0021895 | cerebral cortex neuron differentiation | 1 | 1 | 0.01 | 0.0062 | BP |
| GO:0046958 | nonassociative learning | 1 | 1 | 0.01 | 0.0062 | BP |
| GO:1900136 | regulation of chemokine activity | 1 | 1 | 0.01 | 0.0062 | BP |
| GO:0046833 | positive regulation of RNA export from nucleus | 1 | 1 | 0.01 | 0.0062 | BP |
| GO:0061972 | dermal bone morphogenesis | 2 | 1 | 0.01 | 0.0123 | BP |
| GO:2000179 | positive regulation of neural precursor cell proliferation | 2 | 1 | 0.01 | 0.0123 | BP |
| GO:0035022 | positive regulation of Rac protein signal transduction | 2 | 1 | 0.01 | 0.0123 | BP |
| GO:1903297 | regulation of hypoxia-induced intrinsic apoptotic signaling pathway | 2 | 1 | 0.01 | 0.0123 | BP |
| GO:1990046 | stress-induced mitochondrial fusion | 2 | 1 | 0.01 | 0.0123 | BP |
| GO:0005309 | creatine:sodium symporter activity | 1 | 1 | 0 | 0.0049 | MF |
| GO:0005337 | nucleoside transmembrane transporter activity | 1 | 1 | 0 | 0.0049 | MF |
| GO:0005415 | nucleoside:sodium symporter activity | 1 | 1 | 0 | 0.0049 | MF |
| GO:0008386 | cholesterol monooxygenase (side-chain-cleaving) activity | 1 | 1 | 0 | 0.0049 | MF |
| GO:0016713 | oxidoreductase activity, acting on paired donors, with incorporation or reduction of molecular oxygen, reduced iron-sulfur protein as one donor, and incorporation of one atom of oxygen | 1 | 1 | 0 | 0.0049 | MF |
| GO:0015370 | solute:sodium symporter activity | 78 | 3 | 0.38 | 0.0065 | MF |
| GO:0004725 | protein tyrosine phosphatase activity | 88 | 3 | 0.43 | 0.0091 | MF |
| GO:0001968 | fibronectin binding | 2 | 1 | 0.01 | 0.0097 | MF |
| GO:0005308 | creatine transmembrane transporter activity | 2 | 1 | 0.01 | 0.0097 | MF |
| GO:0047372 | monoacylglycerol lipase activity | 2 | 1 | 0.01 | 0.0097 | MF |
| GO:0045211 | postsynaptic membrane | 2 | 1 | 0.01 | 0.009 | CC |
| GO:0060077 | inhibitory synapse | 2 | 1 | 0.01 | 0.009 | CC |
| GO:0070418 | DNA-dependent protein kinase complex | 2 | 1 | 0.01 | 0.009 | CC |
| GO:0000347 | THO complex | 4 | 1 | 0.02 | 0.018 | CC |
| GO:0045202 | synapse | 49 | 2 | 0.22 | 0.020 | CC |
| GO:0097060 | synaptic membrane | 6 | 1 | 0.03 | 0.027 | CC |
| GO:0016589 | NURF complex | 8 | 1 | 0.04 | 0.035 | CC |
| GO:0005829 | cytosol | 68 | 2 | 0.31 | 0.036 | CC |
| GO:0005765 | lysosomal membrane | 12 | 1 | 0.05 | 0.053 | CC |
| GO:0098852 | lytic vacuole membrane | 12 | 1 | 0.05 | 0.053 | CC |

**Supplementary Table 4: P-values of the two-way PicMin analysis.** Table shows all p-values of the two-way PicMin analysis between all possible pairs of profundal species. The two comparisons of species within lakes (*C. profundus* – *C. albellus* in Lake Thun and *C. nobilis* – *C. muelleri* in Lake Lucerne) are not shown, as they might be biased by sharing of lake specific genomic variation.

|  |  | Lucerne | | Thun | | Constance |
| --- | --- | --- | --- | --- | --- | --- |
|  |  | ***C. nobilis*** | ***C. muelleri*** | ***C. profundus*** | ***C. albellus*** | ***C. gutturosus*** |
| Lucerne | ***C. nobilis*** | - |  |  |  |  |
|  | ***C. muelleri*** | - | - |  |  |  |
| Thun | ***C. profundus*** | 6.8 10^-43^ | 6.7 10^-43^ | - |  |  |
|  | ***C. albellus*** | 7.6 10^-191^ | 1.4 10^-83^ | - | - |  |
| Constance | ***C. gutturosus*** | 1.8 10^-37^ | 3.8 10^-14^ | 2.8 10^-20^ | 2.8 10^-30^ | - |
| Walen | ***C. heglingus*** | 1.0 10^-207^ | 5.2 10-^292^ | 2.4 10^-42^ | 1.6 10^-131^ | 1.5 10^-49^ |
